# Syngas to light olefins conversion with high olefin/paraffin ratio using ZnCrO_x_/AlPO-18 bifunctional catalysts

**DOI:** 10.1038/s41467-019-09336-1

**Published:** 2019-03-21

**Authors:** Junjie Su, Haibo Zhou, Su Liu, Chuanming Wang, Wenqian Jiao, Yangdong Wang, Chang Liu, Yingchun Ye, Lin Zhang, Yu Zhao, Hongxing Liu, Dong Wang, Weimin Yang, Zaiku Xie, Mingyuan He

**Affiliations:** 1 State Key Laboratory of Green Chemical Engineering and Industrial Catalysis, Shanghai Research Institute of Petrochemical Technology, SINOPEC Corp., Shanghai, 201208 China; 2 China Petrochemical Corporation (SINOPEC Group), Beijing, 100728 China; 3 Shanghai Key Laboratory of Green Chemistry and Chemical Processes, School of Chemistry and Molecular Engineering, East China Normal University, Shanghai, 200062 China

## Abstract

Direct synthesis of light olefins from syngas (STO) using a bifunctional catalyst composed of oxide and zeolite has attracted extensive attention in both academia and industry. It is highly desirable to develop robust catalysts that could enhance the CO conversion while simultaneously maintain high selectivity to C2-C4 olefins. Herein, we report a bifunctional catalyst consisting of ZnCr binary oxide (ZnCrO_x_) and low-Si AlPO-18 zeolite, showing both satisfying selectivity to C2-C4 olefins of 45.0% (86.7%, CO_2_ free) and high olefin/paraffin ratio of 29.9 at the CO conversion of 25.2% under mild reaction conditions (4.0 MPa, 390 °C). By optimizing the reaction conditions, the CO conversion could be markedly increased to 49.3% with a slight drop in selectivity. CD_3_CN/CO-FTIR characterizations and theoretical calculations demonstrate that low-Si AlPO-18 zeolite has lower acid strength, and is therefore less reactive toward the hydride transfer in the STO reaction, leading to a higher olefin/paraffin ratio.

## Abstract (web summary)

Developing robust catalysts for direct synthesis of light olefins from syngas remains a challenge. Here, the authors report a novel bifunctional catalyst composed of ZnCr binary oxide and low-Si AlPO-18 zeolite which exhibits enhanced CO conversion and selectivity to C2-C4 olefins under mild reaction conditions.

## Introduction

Light olefins (C2–C4) are very important chemical materials in the whole industrial manufacture chain with huge market demand. In the past decade, methanol to olefins (MTO) conversion, a new technology for producing light olefins, has been successfully industrialized^1–4^. However, to produce olefins from raw materials such as coal or natural gas, many precedent processes including syngas production and methanol synthesis are required before the MTO process, highly increasing investment cost and energy consumption. The direct conversion of syngas into light olefins (STO) is therefore strongly desired since 1980s^1,5,6^.

Great efforts have been devoted to the direct STO conversion using modified Fischer-Tropsch (FT) catalysts^7–13^. But it is generally difficult to achieve high selectivity to C2–C4 hydrocarbons, owing to the limitation of the Anderson–Schulz–Flory (ASF) distribution. Although some modified methods like introducing a second zeolite phase to conventional FT catalysts can optimize the hydrocarbons distribution, the olefin selectivity is still limited^14–18^. More recently, using a bifunctional catalyst containing ZnCr oxide and SAPO-34 zeolite, Bao and coworkers^19^ first reported direct synthesis of light olefins from syngas exhibiting an excellent selectivity of 80% (CO_2_ free) for light olefins at ~17% CO conversion. Inspired by this pioneered work, several kinds of bifunctional catalysts to directly convert CO or CO_2_ into light olefins were then developed, including ZnZr/SAPO-34^20^, ZnAl/SAPO-34^21^, MnO_x_/SAPO-34^22^, and InZr/SAPO-34^23^. Interestingly, as the selectivity of light olefins is close to that of the MTO reaction, this STO process therefore holds the potential to be a promising alternative to directly produce light olefins from syngas.

Despite the great progress made previously, the industrialization of the direct conversion is still impeded by several major challenges. First, the CO conversion should be significantly improved to decrease the energy consumption for the separation of unconverted syngas from light olefins products. Second, the effective utilization of carbon resources is low as the result of undesired CO_2_ production; currently, either modified FT catalysts^7,24^ or bifunctional catalysts^19–23^, both show a CO_2_ selectivity of higher than 45%. However, for the conversion of hydrogen lean syngas derived from coal or biomass, it’s necessary to modulate H_2_/CO ratio via water-gas shift reaction. Consequently, high CO_2_ selectivity can enable the in situ re-adjustment of the H_2_/CO molar ratio, which is benefit for coal- or biomass-based syngas conversion. Third, among the CO_2_-free products, it is very necessary to enhance the selectivity to the desired products of light olefins while reduce the selectivity to paraffins and maintain high CO conversion. The maximal CO conversion of the direct STO process using bifunctional catalysts is reported to be only ~17%^19^. As olefins may be further hydrogenated into paraffins in the presence of hydrogen^23^, the selectivity of C2–C4 paraffins would be increased when enhancing the CO conversion. So it is very challenging to simultaneously achieve high CO conversion and selectivity to light olefins in the direct STO conversion.

We previously found that the olefin/paraffin ratio could be increased by modifying the oxide component with alkali metals to reduce the hydrogenation activity^23^. However, this approach also leads to the decrease of CO conversion. In addition, it was demonstrated that the acidity and/or structures of zeolites can affect the distribution of products. For example, when ZSM-5 with stronger acidity is used as the zeolite component, the primary products of syngas conversion are paraffins^18^ or aromatics^25,26^, in sharp contrast to the product distribution of SAPO-34 zeolites in which high-light olefins selectivity of ~80% (CO_2_ free) and olefin/paraffin ratio of ~5 was achieved. Wang et al.^20^ reported that the olefins selectivity can be slightly enhanced by reducing Si contents in SAPO-34; the maximum olefin/paraffin ratio is <6 at the CO conversion is 11%. These findings imply that tailoring the structure and acidity of zeolites in bifunctional catalysts could be very effective to improve the CO conversion and the olefin selectivity. However, the relationship between structural properties (acidity and cage structure) of zeolites and product distribution in syngas conversion is still unclear, which is closely related to whether the reaction performance, and even whether the industrial competitiveness of this system, can be further improved.

So far, SAPO-34 is usually selected as the candidate of zeolites for C–C coupling to produce light olefins due to its eight-membered ring channels with the size of 3.8 Å × 3.8 Å which is beneficial to the diffusion of light olefins. In this way, no significant enhancement on catalytic performance was observed by altering the oxide component in bifunctional catalysts^19–23^. Different from previous strategy, in this work we design a bifunctional catalyst composed of the ZnCrO_x_ and a zeolite with AEI framework, whose main channel is also eight-membered ring channels with the size of 3.8 Å × 3.8 Å. The bifunctional catalyst containing ZnCrO_x_ and low-Si AlPO-18 zeolite shows an excellent performance, achieving a C2–C4 olefin selectivity of 87% (CO_2_ free) and olefin/paraffin ratio of 29.9 at the CO conversion of 25.2%.

## Results

### Characterization of the catalyst materials

**Fig. 1:**
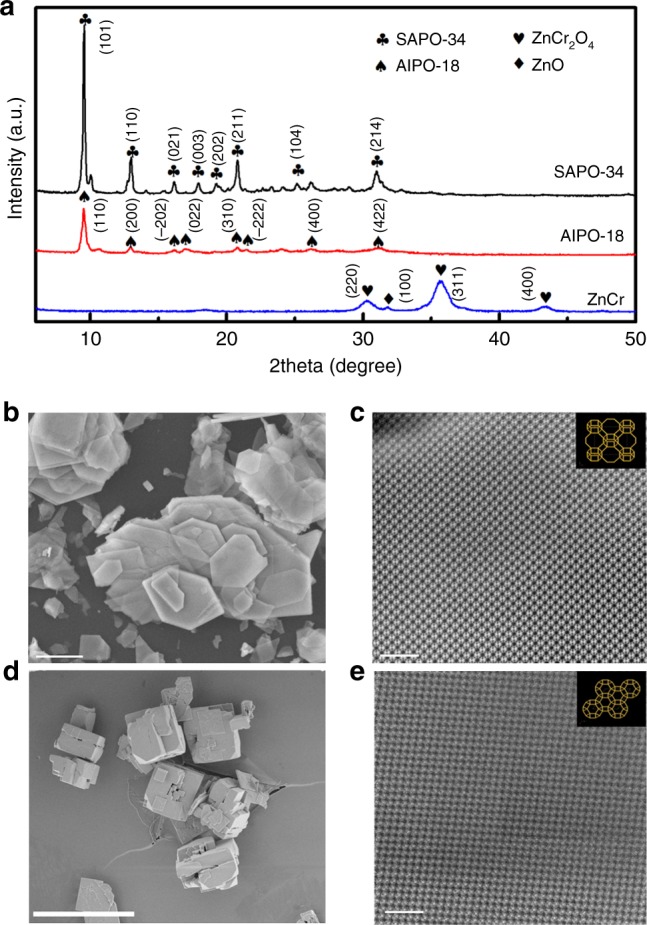
Microstructure of bifunctional catalysts. **a** XRD patterns of the calcined ZnCrO_x_, AlPO-18 and SAPO-34 zeolites, SAPO-34(club), AlPO-18(spade), ZnCr_2_O_4_(heart), and ZnO(diamond). **b** SEM images of the calcined AlPO-18 (scale bar, 1 μm). **c** Aberration-corrected STEM images and the corresponding framework images of the calcined AlPO-18 (scale bar, 5 nm). **d** SEM images of the calcined SAPO-34 (scale bar, 10 μm). **e** Aberration-corrected STEM images and the corresponding framework images of the calcined SAPO-34 (scale bar, 5 nm)

The geometry structures of zeolite components were characterized to shed light on the influence of zeolite structure and acidity on syngas conversion. The XRD patterns in Fig. 1a show that AlPO-18 and SAPO-34 zeolites are typical AEI and CHA topology structure with high crystallinity, respectively. The SAPO-34 zeolite appears as cuboids with crystal size of 2–4 μm (Fig. 1d), which is a typical crystal morphology of the CHA framework (trigonal). The AlPO-18 crystals show nanosheet morphologies with the diameter of 1–4 μm and the thickness of 100 nm (Fig. 1b), typical features of the AEI framework (orthorhombic). Their chemical compositions, BET surface areas and pore volumes of the calcined samples are provided in Table 1. The surface area of calcined AlPO-18 is 592 m^2^ g^−1^, higher than that of SAPO-34 (493 m^2^ g^−1^). There is no obvious difference in the micropore volume, but the total pore volume of AlPO-18 is twice larger than SAPO-34, owing to the increase of mesoporous pores caused by reduced crystal sizes.

**Table 1:** Chemical compositions, BET surface areas and pore volumes of the calcined zeolites

Sample	Si/Al mole ratio^a^	BET surface area^b^ (m^2 ^g^−1^)	Micropore area^b^ (m^2^ g^−1^)	Total pore volume^b^ (cm^3^ g^−1^)	Micropore volume^b^ (cm^3^ g^−1^)
AlPO-18	0	592	501	0.64	0.23
low-Si AlPO-18	0.013	467	384	0.60	0.18
SAPO-34	0.13	493	490	0.27	0.23

^a^Determined by inductively coupled plasma atomic emission spectroscopy

^b^Determined by N_2_-adsorption

Atomic hyperfine structures of two zeolites were also determined using the spherical aberration-corrected STEM, as shown in Fig. 1c, e. We found that AlPO-18 shows eight-membered framework rings originated from the (001) crystal plane, while SAPO-34 mainly exposes the (001) crystal plane with six-membered rings. It was difficult to detect other planes of SAPO-34 with eight-membered rings, such as the (100), (010), and (110), even by adjusting the measurement direction of STEM, indicating that the number of exposed eight-membered ring channels in AlPO-18 is higher than the SAPO-34 zeolite.

### Catalytic performances

**Fig. 2:**
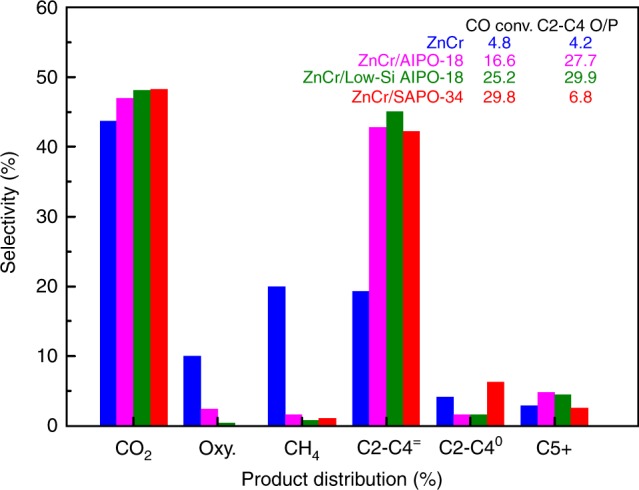
Catalytic performance of bifunctional catalyst. Reaction conditions: 390 °C, 4.0 MPa, 1200 h^−1^, H_2_/CO = 1, OX/ZEO = 1 (Oxy.: oxygenates including ethanol and methyl ether; C2–C4 O/P: C2–C4 olefin/paraffin ratio; C2–C4^=^: C2–C4 olefins; C2–C4^0^: C2–C4 paraffins; C5+: hydrocarbon products with more than five carbon)

We thoroughly investigated and compared the catalytic performance and product distribution of different catalysts (Fig. 2). The significance of zeolite component is demonstrated. Single component of ZnCrO_x_ exhibits very low CO conversion of 4.8% and also low selectivity to olefins (19.0%) at 4.0 MPa, 390 °C. By integrating with zeolites (mass ratio 1:1; granule-stacking), bifunctional catalysts generally show much higher CO conversion (>15%) and light olefins selectivity (>40%) under the same reaction conditions. However, significant differences also exist among those bifunctional catalysts. The ZnCr/AlPO-18 bifunctional catalyst shows a CO conversion of 16.6% with a C2–C4 olefin selectivity of 42.7% (80.6%, CO_2_ free). In addition, a olefin/paraffin ratio of 27.7 is obtained, which is higher than the value of 6.8 for the ZnCr/SAPO-34 catalyst. The lower CO conversion and oxygenate selectivity imply that the driving force of AlPO-18 zeolite is insufficient for this reaction, possibly relevant with two aspects of the particle size and acidity. Regarding the former aspect, we further synthesized an AlPO-18 zeolite with 1–2 μm particle size (AlPO-18-L, Supplementary Fig. 1) to investigate the size effect of zeolite on the catalytic performance (Supplementary Table 1). The ZnCr/AlPO-18-L shows even lower CO conversion (8.8%) and a higher selectivity to oxygenates (DME and MeOH, 30.8%). However, it is noteworthy that although the total selectivity of hydrocarbons decreases, the C2–C4 olefin/paraffin ratio remains at a level of 27.5, which means that the particle sizes of zeolite does not show significant effect on hydrocarbon distribution. In the case of the acidity effect, we synthesized a low-Si AlPO-18 zeolite (Si/Al ratio = 0.013) by using boehmite containing trace Si impurities as Al resource. The ZnCr/low-Si AlPO-18 bifunctional catalyst shows CO conversion of 25.2%, which is close to that of ZnCr/SAPO-34 catalyst. Meanwhile, the selectivity of C2–C4 olefins is increased to 45.0% (86.7% CO_2_ free), while that of C2–C4 paraffins is as low as 1.5%, leading to a C2–C4 olefin/paraffin ratio of 29.9. In addition, it is necessary to note that our results of ZnCr/low-Si AlPO-18 are also markedly better than previously reported catalysts, such as ZnCr/SAPO-34^19^, ZnZr/SAPO-34^20^, MnO_x_/SAPO-34^22^, and InZr/SAPO-34^23^, in the case of olefin/paraffin ratio.

**Fig. 3:**
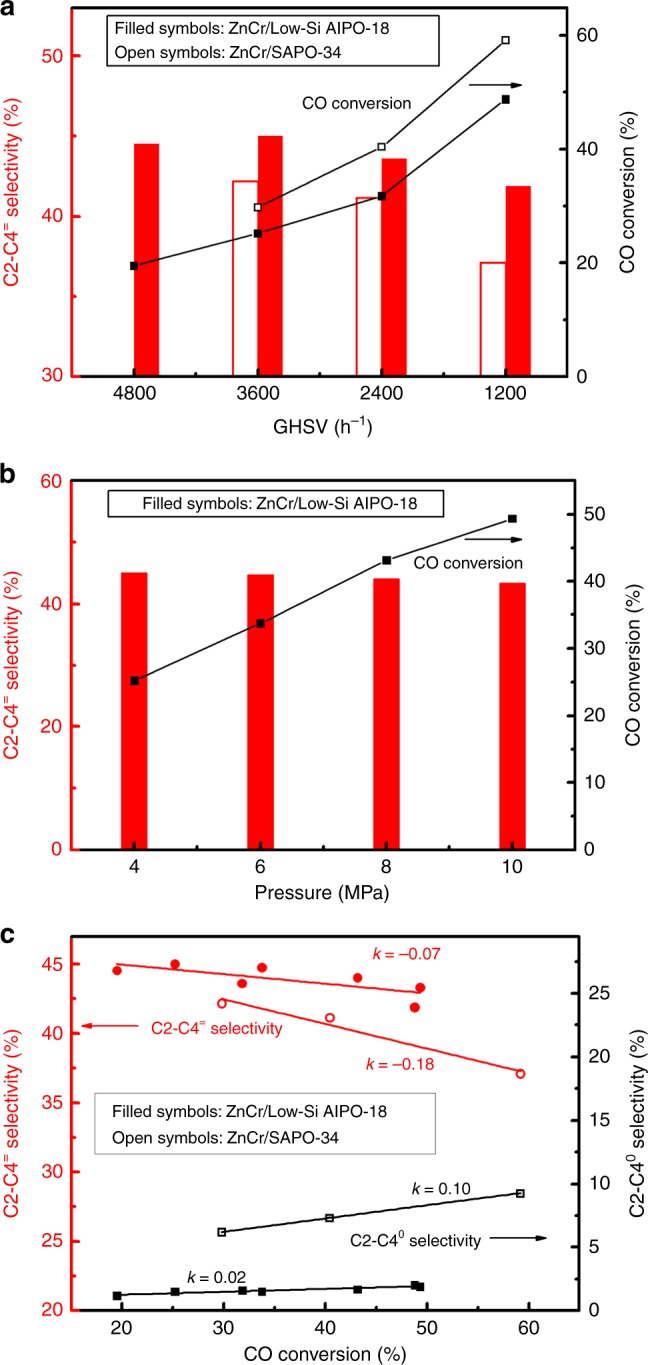
Effect of reaction conditions on catalytic performance. **a** Effect of GHSV on CO hydrogenation over ZnCr/low-Si AlPO-18 and ZnCr/SAPO-34 bifunctional catalysts; Other conditions: 390 °C, 4.0 MPa, H_2_/CO = 1. **b** Effect of pressure on CO hydrogenation over ZnCr/low-Si AlPO-18 bifunctional catalysts; Other conditions: 390 °C, 3600 h^−1^, H_2_/CO = 1. **c** Relation between CO conversion and C2–C4 product selectivity (ZnCr/low-Si AlPO-18: filled symbols, ZnCr/SAPO-34: open symbols)

The influence of reaction conditions, such as the GHSV and reaction pressure, on catalytic performance was then studied. As shown Fig. 3a, the CO conversion of ZnCr/low-Si AlPO-18 and ZnCr/SAPO-34 bifunctional catalysts increases with the decrease of GHSV, while the selectivity of C2–C4 olefins is gradually dropped. As shown in Fig. 3b, we can see that by increasing pressure from 4 to 10 MPa (3600 h^−1^, 390 °C, CO/H_2_ = 1), the CO conversion of ZnCr/low-Si AlPO-18 catalyst is obviously improved from 25.2 to 49.3% while the selectivity to C2–C4 olefins is only slightly decreased from 45.0 to 43.3%. In addition, H_2_/CO mole ratio is another effect on this conversion. As shown in Supplementary Table 3, with an increase in H_2_/CO mole ratio, the CO conversion rises while the H_2_ conversion drops obviously. As high H_2_ concentration may facilitate the olefins hydrogenation and reverse water-gas shift reaction, our results show gradual decrease in the selectivity to C2–C4 olefins and CO_2_ as H_2_/CO increases despite of the increase of carbon utilization (selectivity to hydrocarbons).

From Fig. 3c, it was observed that increasing the CO conversion would inevitably lead to the decrease in C2–C4 olefins selectivity, as depicted by linear relations, but the slope of those trends varies with zeolite component. Obviously, ZnCr/SAPO-34 shows a much sharper declining slope in C2–C4 olefins selectivity than ZnCr/low-Si AlPO-18 when increasing CO conversion. By optimizing reaction conditions, we can achieve a very high CO conversion of more than 50% for the ZnCr/SAPO-34. However, it is difficult to keep the C2–C4 olefin selectivity higher than 80% (CO_2_ free) at the same time, which has also been proved in Bao’s work^19^. On the contrary, using ZnCr/low-Si AlPO-18 catalyst, it is possible to achieve both high C2–C4 olefin selectivity and high CO conversion simultaneously, offering potential for industrial application.

### Structure and performance relationship

We are now at the position to figure out why the performance of ZnCr/AlPO-18 and ZnCr/low-Si AlPO-18 can be obviously improved compared with ZnCr/SAPO-34. Because the same ZnCrO_x_ was used, the change in catalytic behaviors is mainly caused by the zeolite component. After excluding the influence of particle size on hydrocarbon distribution, it is obvious that both SAPO-34 and low-Si AlPO-18 zeolites mainly differ in Si/Al ratio and framework structures as characterized above. It is therefore difficult to make a direct comparison between the two zeolites. We here construct a bridge between SAPO-34 and low-Si AlPO-18 by synthesizing the low-Si AlPO-34 zeolite, which shows similar Si/Al ratio of 0.013 with low-Si AlPO-18 (Supplementary Table 4). By combining with the same ZnCrO_x_, we measured its activity in syngas conversion under the same reaction conditions. The ZnCr/low-Si AlPO-34 bifunctional catalyst shows a C2–C4 olefin selectivity of 84.3% (CO_2_ free) with a CO conversion of 24.9% (Supplementary Table 1). The obtained olefin/paraffin ratio is 10.5, which is slightly higher than that of ZnCr/SAPO-34 (6.8) while much lower than that of ZnCr/low-Si AlPO-18 (29.9).

**Fig. 4:**
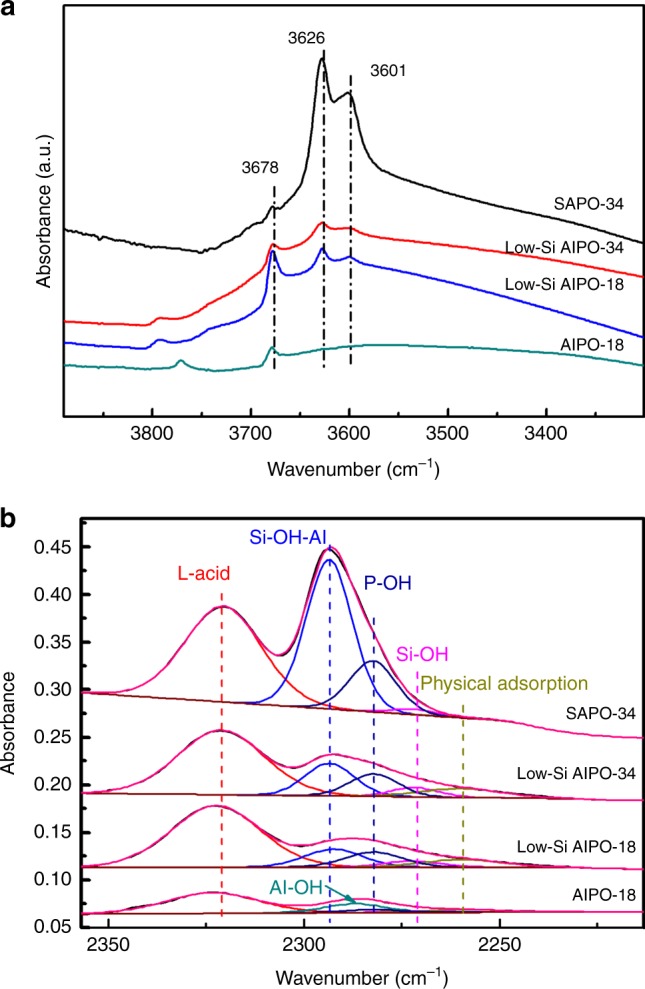
FTIR spectra. **a** FTIR spectra of calcined low-Si AlPO-18, low-Si AlPO-34, and SAPO-34 zeolites after vacuum dehydration at 400 °C. **b** Fitted FTIR spectra of CD_3_CN adsorption over calcined AlPO-18, low-Si AlPO-18, low-Si AlPO-34, and SAPO-34 zeolites at room temperature

Because SAPO-34 and low-Si AlPO-34 have the same CHA framework topology structure, the differences in catalytic performance are largely caused by zeolite acidity. FTIR spectra were applied to characterize the acidity of zeolite, using CD_3_CN or CO as probe molecules^27,28^. As shown in Fig. 4a, three peaks at 3678, 3626, and 3601 cm^−1^ are observed in the FTIR spectra of calcined zeolites after vacuum dehydration at 400 °C, corresponding to different types of hydroxyl vibrations. The peak at 3678 cm^−1^ is assigned to Al–OH or P–OH, the other two peaks are assigned to the HF(3626 cm^−1^) and LF(3601 cm^−1^) Si–OH–Al bridging hydroxyl groups^28,29^, known as Brønsted acid sites in SAPO zeolites. Compared with SAPO-34, the total areas of two Si–OH–Al peaks for low-Si AlPO-34 zeolites are obviously smaller, indicating that the total concentration of Brønsted acid sites (acid density) in low-Si AlPO-34 is much lower than SAPO-34. Similar results are also obtained using in situ CD_3_CN-FTIR (Brønsted acid sites, 2293 cm^−1^, Fig. 4b). Interestingly, previous work^30^ has shown that the isolation of Brønsted acid sites is beneficial to maximize olefin selectivity by preventing secondary reactions in MTO process. In this aspect, the decrease of acid density also helps to promote the acid site isolation. Considering the catalytic difference in SAPO-34 and low-Si AlPO-34 zeolites, we demonstrated that reducing the acid density of zeolites and/or promoting the isolation of acid sites is beneficial to slightly increase the olefin/paraffin ratio, consistent with previous work^19,20,30^.

Another key parameter of zeolite acidity is the acid strength, which may also affect on catalytic performance. As the Si/Al ratio of low-Si AlPO-18 and low-Si AlPO-34 was similar, no obvious difference in acid amount is observed in the FTIR spectra 3630–3590 cm^−1^ region (Fig. 4a)^28^. However, CD_3_CN-FTIR spectra (Fig. 4b) show that there exists slight shift in peak positions ascribed to Brønsted acid sites for low-Si AlPO-34 (2293 cm^−1^) and low-Si AlPO-18 (2287 cm^−1^). Fitting results show that there are two kinds of Brønsted acid sites: Si–OH–Al bridging hydroxyl groups (2293 cm^−1^), and P–OH (2282 cm^−1^), respectively, where the acid strength of P–OH is weaker than that of Si–OH–Al hydroxyl group. In addition, according to the quantified results (Supplementary Table 3), the acid density in low-Si AlPO-18 was similar with low-Si AlPO-34 but the acid strength of low-Si AlPO-34 is stronger than the low-Si AlPO-18. No peaks assigned to Si–OH–Al bridging hydroxyl groups is observed from FTIR spectrum of the AlPO-18 zeolites(Fig. 4a), but there also exists a broad peak at 2287 cm^−1^ ascribed to Brønsted acid sites composed of P–OH or Al–OH.

**Fig. 5:**
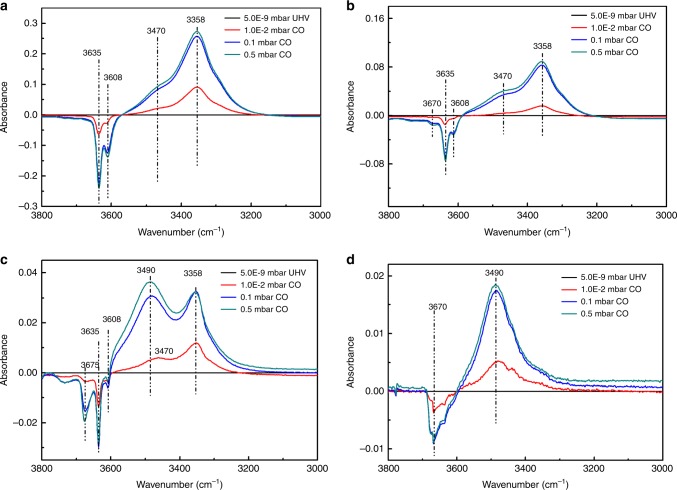
In situ FTIR spectra of CO adsorption. **a** SAPO-34; **b** low-Si AlPO-34; **c** low-Si AlPO-18; and **d** AlPO-18 (UHV: ultra-high vacuum)

In situ CO-FTIR was also employed to further determine the acidity difference between low-Si AlPO-34 and low-Si AlPO-18 zeolites (Fig. 5). After CO adsorption, the peak assigned to HF Si–OH–Al hydroxyl group in low-Si AlPO-34 shifts from 3635^1^ to 3358 cm^−1^ (difference 227 cm^−1^). The FTIR spectrum of low-Si AlPO-18 zeolite at low CO adsorption pressure (5.0E−9 mbar) is similar to that of low-Si AlPO-34. But under higher CO adsorption pressure, an additional OH peak shifts from 3675 cm^−1^ to 3490 cm^−1^ (difference 185 cm^−1^), assigned to P–OH group^28^ and the same shifts are also observed for AlPO-18 zeolite. The smaller shift wavenumber indicates that the acid strength of P–OH is weaker than that of Si–OH–Al hydroxyl group. In addition, the peak intensity of the P–OH is close to the Si–OH–Al hydroxyl group, and thus demonstrates that P–OH is one of the main sources of Brønsted acid sites in AlPO-18 and low-Si AlPO-18 zeolites. These results show that, despite of difference in pore structures, the weaker acid strength should be one of main reasons for the obvious increase of olefin/paraffin ratio over bifunctional catalyst containing AlPO-18 or low-Si AlPO-18 zeolite.

**Fig. 6:**
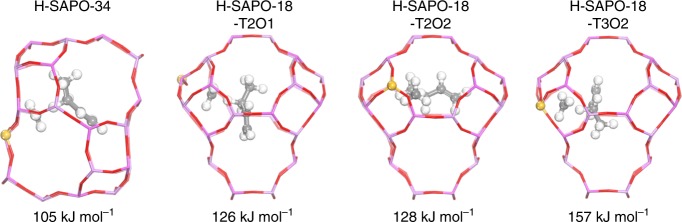
Hydride transfer barrier. Calculated hydride transfer barrier between methoxide and 1-butene in H-SAPO-34 and H-SAPO-18 zeolites

In the bifunctional catalysts for the direct conversion of syngas to light olefins, methanol, and/or ketene were previously suggested as the key intermediate produced on oxides and transformed in zeolites to olefins^19,20^. We have theoretically proved that the ketene to olefins conversion proceeds via the hydrocarbon pool mechanism, similar to the MTO conversion in zeolites where CO or H_2_O were, respectively, formed in both cycles^31^. Therefore, the selectivity of olefins mainly correlates with the distribution of cracking precursors, highly affected by the structure of zeolites (e.g., acidity, topology) and reaction conditions^2,32^. It was found that the hydride-transfer reaction between two olefins highly contributes to the formation of alkanes in Brønsted acid zeolites, and which should be suppressed to achieve high olefin/paraffin ratio. Periodic density functional theory (DFT) calculations were performed to understand the effect of zeolite framework on the activity of the hydride transfer, and finally on the distribution of paraffins. The hydride transfer between methoxide and 1-butene was used as the model reaction. The enthalpy barrier at 0 K was calculated to be 105 kJ mol^−1^ in H-SAPO-34, much lower than the results in H-SAPO-18 in which several different acidic sites were considered (126~157 kJ mol^−1^, see Fig. 6). These results indicated that H-SAPO-18 is less reactive than H-SAPO-34 for the hydride transfer, leading to a relatively higher olefin/paraffin ratio in AEI-structured zeolites as observed in experiments. Regarding the intermediate species, we speculated that methanol is likely to play a greater role than ketene, since relatively larger amounts of oxygenates (methanol or DME) are produced on pure ZnCrO_x_ and ketene is less reactive than methanol for the chain propagation in the hydrocarbon pool mechanism^31,33^.

### Stability of bifunctional catalyst

**Fig. 7:**
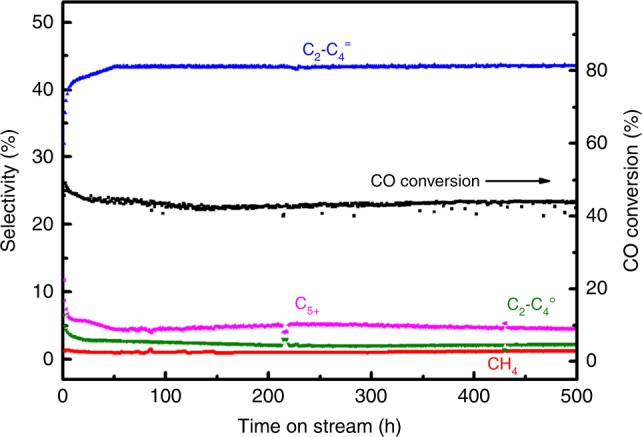
Stability of catalyst. Time-on-stream of CO conversion and selectivity for ZnCr/low-Si AlPO-18 bifunctional catalyst. Reaction conditions: 390 °C, 4.0 MPa, 1200 h^−1^, H_2_/CO = 1, and oxide/zeolite mass ratio = 1. (C2–C4^=^: C2–C4 olefins; C2–C4^0^: C2–C4 paraffins; C5 + : hydrocarbon products with more than five carbon)

Finally, we can rationalize the excellent catalytic performance of ZnCr/low-Si AlPO-18 catalyst from the points of acid strength, acid density, and framework structures of zeolite component. In addition, the catalyst stability is also a key indicator in industrial applications, and thus we tested the stability of ZnCr/low-Si AlPO-18 catalyst for 500 h under reaction conditions of 390 °C, 4.0 Mpa, 1200 h^−1^, and H_2_/CO = 1. As shown in Fig. 7, CO conversion keeps stable at around 45%, meanwhile, C2–C4^=^ selectivity maintains at the value of 43% and the olefin/paraffin ratio is always ~20. The TGA curves (Supplementary Fig. 2) of used catalysts shows that there are 1.5 and 15% weight loss in ZnCrO_x_ and low-Si AlPO-18 zeolite, respectively, which contain volatile species and occluded organic deposits. After removal of volatile components in N_2_ flow, 3.5% of carbon deposit amount is obtained by measuring the amount of CO_2_ formed from the combustion of the used bifunctional catalyst. Therefore, ZnCr/low-Si AlPO-18 is a highly potential candidate of bifunc*t*ional catalyst for the direct synthesis of light olefins from syngas with high olefin/paraffin ratio and superior stability.

## Discussion

In summary, a series of zeolites with AEI framework were synthesized and were employed to catalyze the direct conversion of syngas to light olefins combining with ZnCrO_x_. Compared with the bifunctional catalysts containing zeolites with CHA framework, ZnCr/low-Si AlPO-18 catalyst shows an excellent catalytic performance with high CO conversion and light olefins selectivity in direct conversion of syngas with low H_2_/CO ratio (1:1). The selectivity to C2–C4 olefins reaches up to 45.0% (86.7%, CO_2_ free) with an olefin/paraffin ratio of 29.9 at a CO conversion of 25.2%. By optimizing the reaction conditions, even higher CO conversion of 49.3% with a C2–C4 olefins selectivity of 43.3% (83.4%, CO_2_ free) and olefin/paraffin ratio of 22.5 is achieved. The catalyst is found to exhibit high stability; no obvious decline in performance was observed after the time-on-stream of 500 h. FTIR characterization approaches and DFT calculations demonstrated that low-Si AlPO-18 zeolite has low acid density and strength and is less reactive for the hydride transfer compared with low-Si AlPO-34 and SAPO-34 zeolites, leading to a higher selectivity to light olefins. Our results pave the way for developing improved catalysts for the industrialization of direct light olefins synthesis from syngas.

## Methods

### Synthesis of the ZnCrO_x_

ZnCrO_x_ was prepared via a co-precipitation method. Analytical grade chemical reagents were used in the experiment without any further purification. Typically, appropriate amount of zinc nitrate (Zn(NO_3_)_2_·6H_2_O; bought from Sinopharm Chemical Reagent Co.) and chromium nitrate (Cr(NO_3_)_4_·9H_2_O; Sinopharm Co.) were dissolved in deionized water to form a mixed salt solution (the total concentration of metal ion is 1.0 mol L^−1^). Meanwhile, ammonium carbonate anhydrous ((NH_4_)_2_CO_3_; Sinopharm Co.) was dissolved in deionized water, resulting in a basic solution (1.0 mol L^−1^) as precipitant. And then, these two solutions were simultaneously added, dropwise, into a beaker containing 20 mL deionized water under continuous stirring at 70 °C. The pH value of the aqueous solution was kept at 7.0–8.0 by adjusting the addition rate of basic solution. After stirring for 2 h at 70 °C, the precipitate was obtained via filtration and washing with deionized water. After dried at 80 °C for 12 h, the samples were then calcined in a muffle furnace at 500 °C for 1 h with a ramping rate of 2 °C min^−1^ under static air. The obtained binary oxides were denoted as ZnCr.

### Synthesis of the zeolites

AlPO-18 was synthesized as the following: 24.5 g aluminum isopropoxide (Al_2_O_3_, 74.6%) was added into the mixture prepared by combining 15.0 g of H_3_PO_4_ (85%) and 36.3 g of water, stirred until homogeneous. Then, 49 g of TEAOH (40%) was added and the mixture was further stirred until homogeneous. The composition of the final reaction mixture in molar oxide ratios was 0.9Al_2_O_3_:P_2_O_5_:(TEA)_2_O:60 H_2_O:5.4 i-C_3_H_7_OH. The reaction mixture was sealed in a stainless steel pressure vessel lined with an inert plastic material (polytetrafluoroethylene) and heated in an oven at 170 °C at autogenous pressure for 48 h. The solid reaction product was recovered by filtration, washed with water, and dried at 100 °C over night, followed by calcination at 550 °C for 4 h with a heating rate of 2 °C min^−1^ to remove the occluded organic species.

Low-Si AlPO-18 was synthesized as the following: 27.4 g boehmite (Al_2_O_3_, 74.6%) was added into the mixture prepared by combining 46.1 g of H_3_PO_4_ (85%) and 119.2 g of water, stirred until homogeneous. Then, HCl solution (37%) and TEAOH (25%) were added and the mixture was further stirred until homogeneous. The composition of the final reaction mixture in molar oxide ratios was 0.33HCl:0.67(TEA)_2_O:Al_2_O_3_:P_2_O_5_:40H_2_O. The reaction mixture was sealed in a stainless steel pressure vessel lined with an inert plastic material (polytetrafluoroethylene) and heated in an oven at 170 °C at autogenous pressure for 72 h. The solid reaction product was recovered by filtration, washed with water, and dried at 100 °C over night, followed by calcination at 550 °C for 5 h with a heating rate of 2 °C min^−1^ to remove the occluded organic species.

AlPO-18-L was synthesized as the following: 27.2 g aluminum isopropoxide (Al_2_O_3_, 74.6%) was added into the mixture prepared by combining 15.0 g of H_3_PO_4_ (85%) and 36.3 g of water, stirred until homogeneous. Then, 49 g of TEAOH (40%) was added and the mixture was further stirred until homogeneous. The composition of the final reaction mixture in molar oxide ratios was 1.0Al_2_O_3_:P_2_O_5_:(TEA)_2_O:60 H_2_O:5.4 i-C_3_H_7_OH. The reaction mixture was sealed in a stainless steel pressure vessel lined with an inert plastic material (polytetrafluoroethylene) and heated in an oven at 200 °C at autogenous pressure for 96 h. The solid reaction product was recovered by filtration, washed with water, and dried at 100 °C over night, followed by calcination at 550 °C for 4 h with a heating rate of 2 °C min^−1^ to remove the occluded organic species.

Low-Si AlPO-34 was synthesized as the following: 27.4 g boehmite (Al_2_O_3_, 74.6%) was added into the mixture prepared by combining 46.1 g of H_3_PO_4_ (85%) and 119.2 g of water, stirred until homogeneous. Then, morpholine and HF were added in succession with the composition of the final reaction mixture in molar oxide ratios being 0.25HF:6.0Mor.:1.0Al_2_O_3_:1.0P_2_O_5_:100H_2_O. The resultant mixtures were stirred for 1 h at room temperature and then was sealed in a stainless steel pressure vessel lined with an inert plastic material (polytetrafluoroethylene) and heated in an oven at 200 °C at autogenous pressure for 72 h. The solid reaction product was recovered by filtration, washed with water, and dried at 100 °C over night, followed by calcination at 550 °C for 5 h with a heating rate of 2 °C min^−1^ to remove the occluded organic species.

SAPO-34 was synthesized as the following: 27.4 g boehmite (Al_2_O_3_, 74.6%) was added into the mixture prepared by combining 46.1 g of H_3_PO_4_ (85%) and 119.2 g of water, stirred until homogeneous. Then, triethylamine (TEA) was added into the mixture with the composition of the final reaction mixture in molar oxide ratios being 0.15SiO_2_:1.0Al_2_O_3_:0.8 P_2_O_5_:3.0TEA:50H_2_O. The resultant mixtures were stirred for 1 h at room temperature and then was sealed in a stainless steel pressure vessel lined with an inert plastic material (polytetrafluoroethylene) and heated in an oven at 200 °C at autogenous pressure for 24 h. The solid reaction product was recovered by filtration, washed with water, and dried at 100 °C over night, followed by calcination at 550 °C for 5 h with a heating rate of 2 °C min^−1^ to remove the occluded organic species.

### Preparation of bifunctional catalysts

For the preparation of the bifunctional catalysts, the ZnCrO_x_ and the zeolites were pressed, crushed, and sieved to granules in the range of 20–40 mesh, respectively. Then, the granules of the two samples were mixed together by shaking in a vessel.

### Catalyst characterization

XRD patterns were obtained using Bruker D8 Advance diffractometer, with an accelerated voltage of 40 kV and detector current of 200 mA. Cu-K_α_ radiation (*λ* = 51.540589 Å) was used for a continuous scanning with the step-size of 0.02° over a 2*θ* range of 5–60°. The crystal size of oxide particles was calculated with the width of diffraction patterns, referring to the full width of half maximum (FWHM) of crystalline facets at (311) for ZnCr_2_O_4_ using Debye–Scherrer formula.

N_2_ Adsorption and desorption isotherms were collected on Micromeritics TriStar3000 at 75 K. Prior to the measurements, the sample was degassed at 350 °C until a stable vacuum of about 0.67 Pa was reached.

The chemical compositions of the calcined samples were determined by inductively coupled plasma atomic emission spectroscopy.

FE-SEM (Field Emission Scanning Electron Microscopy) analysis was performed on a Hitachi S4800 electron microscope with an accelerating voltage of 2.0 kV.

TEM images were conducted on a Tecnai 20 STWIN electron microscope operated at 200 kV. The aberration-corrected scanning transmission electron microscopy (STEM) measurements were performed on a FEI Titan Cubed Themis G2 300 kV with an accelerating voltage of 300 kV. The prepared samples were ultrasonically dispersed in hexane solvent during the ultrasonic treatment and then were dried over a carbon film supported on a Cu grid.

In situ CO-FTIR spectra were recorded using a FTIR spectrometer (Bruker Vertex 70V) equipped with a stainless steel high vacuum transmission infrared cell. The zeolite samples were pressed into spots on a tungsten mesh support. Prior to the experiment, the samples were heated to 350–400 °C with a ramping rate of 12 °C min^−1^ in vacuum (<2.0 × 10^−8^ mbar) for 2.5 h. After cooling down to −150 °C by using liquid nitrogen, the background spectrum was collected. Subsequently, 1 × 10^−2^, 0.1, and 0.5 mbar of CO was introduced into the cell, and then the corresponding spectra were collected at −150 °C.

In situ CD3CN-FTIR spectra were recorded using the same infrared system of CO-FTIR. After dehydrated at 350–400 °C in vacuum (<2.0 × 10^−8^ mbar) for 2.5 h, the samples were cooled down to room temperature, and then the background spectrum was collected. Subsequently, 0.5 mbar of CD_3_CN was introduced into the cell, remaining for 1 h. Finally, CD_3_CN was removed and the corresponding spectra were collected in vacuum.

The amounts of occluded hydrocarbons after the CO hydrogenation reaction were determined by thermogravimetric analysis on a Rigaku standard type thermogravimetric analyzer. In a typical measurement, 0.1 g of sample was heated in an Al_2_O_3_ crucible with a constant heating rate of 10 K min^−1^ and under air purging with a flow rate of 30 mL min^−1^.

The amounts of carbon deposits in used zeolites were determined on a HIR-944C infrared carbon and sulphur analyzer. Prior to the experiment, the sample was first pretreated at 500 °C in N_2_ (60 mL min^−1^) at 0.1 MPa with a ramping rate of 2 °C min^−1^.

### Catalyst evaluation

To assess the catalytic performance, experiments were carried out using a continuous stainless steel fixed-bed micro-reactor lined with a quartz tube (inner diameter, 6 mm). Catalyst bed was located in the middle of the reactor. All feed gas (H_2_ and syngas) were pre-purified in a trap to remove carbonyl compounds at 140 °C. Prior to the reaction, the catalyst was first reduced at 400 °C in H_2_ (100 mL min^−1^) at 0.1 MPa with a ramping rate of 4 °C min^−1^. After pretreatment, the reactor was pressurized using H_2_/CO/N_2_ (45/45/10) to reach the reaction pressure. Influent and effluent gases were analyzed via an online gas chromatography (Agilent-7890B). A capillary column (HP-Plot Q) connected to a flame ionization detector (FID) was used to analyze all products (hydrocarbons and oxygenates). Other gas products were analyzed using a TDX-01 packed column connected with a thermal conductivity detector (TCD). Each data for computing the reaction kinetics was acquired after 20 h. CO conversion and CO_2_ selectivity were determined using an internal standard, and the selectivity of the carbon-containing products was calculated by an internal normalization method. The CO conversion and selectivity are defined in supplementary methods

### Computational methods and modeling

All periodic DFT calculations were carried out using the Vienna Ab initio Simulation Package (VASP 5.3.5)^34^. The Bayesian error estimation functional with vdW correlation (BEEF-vdW) was employed^35^. The electron–ion interaction was described by the projector augmented wave (PAW) method^36,37^. Further details on calculation are given in the Supporting methods.

## Supplementary information

Supplementary Information

Peer Review File

## Data Availability

The authors declare that the data supporting the findings of this study are available within the article and supplementary information file, or from the corresponding author upon reasonable request.
