# Supplementary material for: Syngas to light olefins conversion with high olefin/paraffin ratio using ZnCrOx/AlPO-18 bifunctional catalysts
Source: Nat Commun. 2019 Mar 21;10:1297. doi: 10.1038/s41467-019-09336-1 (PMC6428864; doi:10.1038/s41467-019-09336-1)
Supplement: Supplementary file 1 — Supplementary Information [file 41467_2019_9336_MOESM1_ESM.pdf]

Supporting Information

Syngas to light olefins conversion with high olefin/paraffin ratio using ZnCrOx/AlPO-18 bifunctional catalysts

Su et al.

Supplementary Methods

Calculation

The calculation of conversion and selectivity is given below:

CO conversion (X_{CO}): $$X_{CO} = \frac{CO_{in} - CO_{out}}{CO_{in}} \times 100\% \quad (1)$$

CO_2 selectivity (S_{CO2}): $$S_{CO\,2} = \frac{CO2_{out}}{CO_{in} - CO_{out}} \times 100\% \quad (2)$$

The selectivity of hydrocarbon product C_nH_m (S_{CnHm}):

$$S_{CnHm} = \frac{nC_nH_{m\ out}}{CO_{in} - CO_{out}} \times 100\% \quad (3)$$

where, CO_{in} represents for moles of CO at the inlet gases, CO_{out} for moles of CO at the outlet gases, $CO_{2\ out}$ for moles of CO_2 at the outlet gases, and $C_nH_{m\ out}$ for moles of hydrocarbon products C_nH_m at the outlet gases; n is the carbon number of C_nH_m component.

Computational methods and modeling

All periodic DFT calculations were carried out using the Vienna Ab initio Simulation Package (VASP 5.3.5).[1] The Bayesian error estimation functional with vdW correlation (BEEF-vdW) was employed. [2] The electron-ion interaction was described by the projector augmented wave (PAW) method. [3,4] The plane wave basis set kinetic energy cutoff was 400 eV. The sampling of Brillouin zone was only with Γ point. The dimer method was utilized to locate transition states. [5] A force threshold of 0.01 eV/Å was used for structure optimization of all intermediates and transition states. The frequency calculations employing a partial Hessian approach was used to calculate zero point energies (ZPE).

The H-SAPO-34 and H-SAPO-18 were represented by periodic 36T and 48T cells, respectively. The lattice constants were optimized using 800 eV energy cutoff and 0.01 eV/Å force threshold (13.89, 13.89, 15.09 Å for AlPO-34, and 13.91, 12.87, 18.79 Å for AlPO-18). All atoms were allowed to relax in the calculations while the lattice constants were fixed at the optimized values.

Supplementary Table 1. Catalytic performance of bifunctional catalyst containing ZnCr oxide and different zeolites.

Catalyst	CO conv. (%)	H_2 conv. (%)	Selectivity (%)			Hydrocarbon distribution[c] (%)				C_{2-4} O/P ratio
			CO_2	HC.	Oxy. [d]	CH_4	$C_{2-4}^{=}$	C_{2-4}^{0}	C_{5+}	
ZnCr[a]	4.8	4.4	43.7	46.3	10.0	43.0	41.0	9.7	6.3	4.2
ZnCr/AlPO-18[b]	16.6	10.3	47.0	50.6	2.4	3.0	84.4	3.0	9.6	27.7
ZnCr/low-Si AlPO-18[b]	25.2	14.6	48.1	51.6	0.3	1.4	86.7	2.9	8.9	29.9
ZnCr/low-Si AlPO-34[b]	24.9	14.5	48.2	51.8	0.0	1.9	84.4	7.9	5.8	10.5
ZnCr/SAPO-34[b]	29.8	17.6	48.2	51.8	0.0	1.9	81.5	12.0	4.6	6.8
ZnCr/AlPO-18-L[b]	8.8	7.2	39.9	29.3	30.8	3.4	86.0	3.1	7.5	27.5

[a] reaction conditions: 390 ℃, 4.0MPa, 1200h^{-1}, H_2/CO = 1, OX/ZEO=1; [b] reaction conditions: 390 ℃, 4.0MPa, 3600h^{-1}, H_2/CO ratio = 1, OX/ZEO=1; [c] Hydrocarbon distribution was calculated based on carbon molar amount without CO_2 and Oxygenates; [d] include methanol and methyl ether.

Supplementary Table 2. Catalytic performance of ZnCr/ low-Si AlPO-18 and ZnCr/SAPO-34 bifunctional catalysts under different reaction conditions [a].

Catalyst	Reaction conditions	CO conv. (%)	Selectivity (%)			Hydrocarbon distribution[b] (%)				C_{2-4} O/P ratio
			CO_2	HC.	Oxy. [c]	CH_4	$C_{2-4}^{=}$	C_{2-4}^{0}	C_{5+}	
ZnCr/low-Si AlPO-18	4 Mpa, 1200 h^{-1}	48.8	47.2	52.8	0.1	1.3	80.7	4.0	14.0	20.7
	4 Mpa, 2400 h^{-1}	31.8	48.8	51.0	0.2	1.2	84.0	3.1	11.7	27.1
	4 Mpa, 3600 h^{-1}	25.2	48.1	51.6	0.3	1.4	86.7	2.9	8.9	29.9
	4 Mpa, 4800 h^{-1}	19.5	49.7	49.8	0.5	1.2	85.8	2.3	10.7	37.3
	6 Mpa, 3600 h^{-1}	33.8	49.0	50.7	0.3	1.4	86.2	2.9	9.5	29.7
	8 Mpa, 3600 h^{-1}	43.2	49.5	50.2	0.3	1.4	84.8	3.3	10.4	26.0
	10 Mpa, 3600 h^{-1}	49.3	48.6	51.0	0.4	1.5	83.4	3.7	11.1	22.5
ZnCr/SAPO-34	4 Mpa, 1200 h^{-1}	59.2	45.3	54.7	0	1.7	71.6	17.7	9.0	4
	4 Mpa, 2400 h^{-1}	40.4	47.6	52.4	0	1.9	79.4	14.2	4.5	5.6
	4 Mpa, 3600 h^{-1}	29.8	46.1	53.9	0	1.9	81.5	12.0	4.6	6.8

[a] reaction conditions: 390 ℃, H_2/CO = 1, OX/ZEO=1; [b] Hydrocarbon distribution was calculated based on carbon molar amount without CO_2 and Oxygenates; [c] include methanol and methyl ether.

Supplementary Table 3. Effect of H_2/CO on CO hydrogenation over ZnCr/ low-Si AlPO-18 bifunctional catalyst [a]

H_2/CO	CO conv. (%)	H_2 conv. (%)	Selectivity (%)			Hydrocarbon distribution[b] (%)				C_{2-4} O/P ratio
			CO_2	HC.	Oxy. [c]	CH_4	$C_{2-4}^{=}$	C_{2-4}^{0}	C_{5+}	
0.5	29.5	39.9	45.0	54.7	0	1.5	82.5	2.2	13.8	39.3
1.0	48.8	29.9	47.2	52.6	0.1	1.3	80.7	4.0	14.0	20.7
2.0	68.7	26.7	42.0	58	0	1.4	77.2	7.8	13.4	10.0
4.0	70.7	16.3	38.2	61.8	0	2.4	67.5	16.5	13.6	4.1

[a] reaction conditions: 390 ℃, 4.0MPa, $1200h^{-1}$, OX/ZEO=1; [b] Hydrocarbon distribution was calculated based on carbon molar amount without CO_2 and Oxygenates; [c] include methanol and methyl ether.

Supplementary Table 4. Chemical compositions, BET surface areas and pore volumes of the calcined low-Si AlPO-34 zeolites.

Sample	Si/Al mole ratio	surface area (m^2/g)		Pore volume(cm^3/g)	
		BET surface area	micropore area	total pore	micropore
Low-Si AlPO-34	0.013	358	229	0.55	0.11
AlPO-18-L	0	546	534	0.28	0.24

Supplementary Table 5. Quantified data of fitted CD3CN adsorption FTIR spectra for calcined low-Si AlPO-18, low-Si AlPO-34 and SAPO-34 zeolites.

Sample	SAPO-34		low-Si AlPO-34		low-Si AlPO-18		AlPO-18	
	Position /cm^{-1}	area	Position /cm^{-1}	area	Position /cm^{-1}	area	Position /cm^{-1}	area
L-acid	2321	2.52	2321	1.91	2322	1.89	2323	0.67
Si-OH-Al	2294	2.36	2293	0.55	2292	0.37	2292	0
P-OH	2282	0.81	2282	0.38	2282	0.31	2282	0.06
Al-OH	2287	0	2287	0	2287	0	2287	0.26
Si-OH	2272	0.08	2272	0.15	2273	0.13	2272	0.03
Pysical adsorpution	2261	0.01	2260	0.25	2260	0.22	2260	0.08

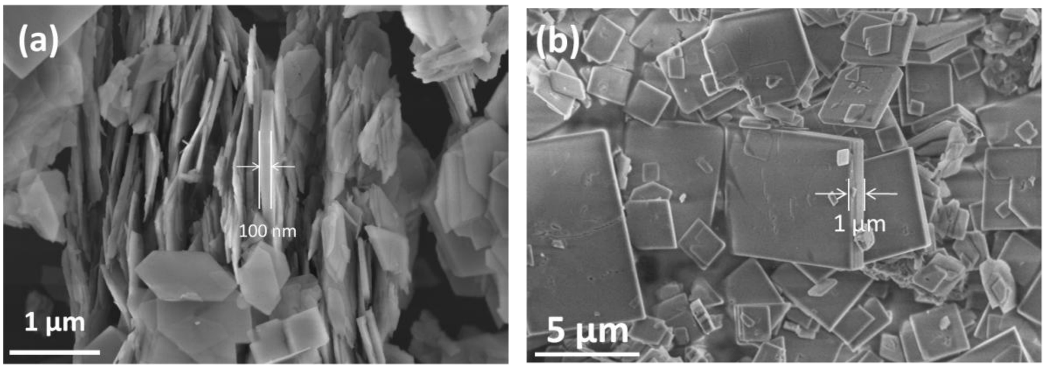

Supplementary Figure 1. SEM images of the calcined AlPO-18(a) and AlPO-18-L(b).

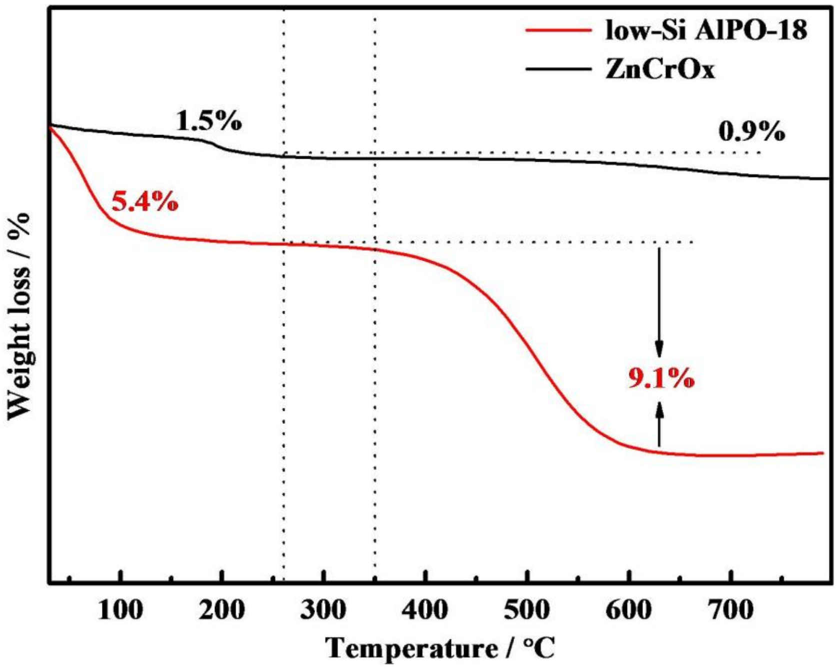

Supplementary Figure 2. TGA curves of used ZnCrOx oxide and low-silica AlPO-18 zeolte obtained after syngas conversion at 673 K.

Supplementary References

[1] G. Kresse, J. Furthmuller, Efficient iterative schemes for *ab initio* total-energy calculations using a plane-wave basis set, *Physical Review B*, **54 (**1996), 11169-11186.

[2] J. Wellendorff, K. T. Lundgaard, A. Møgelhøj, V. Petzold, D. D. Landis, J. K. Nørskov, T. Bligaard, K. W. Jacobsen, Density Functionals for Surface Science: Exchange-correlation Model Development with Bayesian Error Estimation, *Physical Review B*, **85 (**2012), 235149.

[3] P. E. Blochl, Projector Augmented-wave Method, *Physical Review B*, **50** (1994), 17953-17979.

[4] G. Kresse, D. Joubert, From Ultrasoft Pseudopotentials to the Projector Augmented-wave Method, *Physical Review B*, **59 (**1999), 1758-1775.

[5] Graeme Henkelman, Hannes Jónsson, A Dimer Method for Finding Saddle Points on High Dimensional Potential Surfaces Using Only First Derivatives, *The Journal of Chemical Physics*, **111** (1999), 7010-7022.
