# Supplementary material for: Syngas to light olefins conversion with high olefin/paraffin ratio using ZnCrOx/AlPO-18 bifunctional catalysts
Source: Nat Commun. 2019 Mar 21;10:1297. doi: 10.1038/s41467-019-09336-1 (PMC6428864; doi:10.1038/s41467-019-09336-1)
Supplement: Supplementary file 2 — Peer Review File [file 41467_2019_9336_MOESM2_ESM.pdf]

Reviewers' comments:

Reviewer #1 (Remarks to the Author):

This is an interesting manuscript that may have potential for Nature Communications but not in its current form.

First of all, the manuscript is misleading: all selectivities reported are in a CO_2 free basis, considering that CO_2 selectivities (only reported in the caption of figure 1) are in the order of a 50%, this means that selectivities to all the other products are actually half of those reported. When put in perspective, catalytic results are not far from those obtained when doing classical FTS chemistry on K promoted Fe catalysts (see for instance Nature Commun. 6 (2015) 6451). This is in principle fine, with CO:H_2 ratios of 1, one would want to make CO_2 to account for the H_2 poor feed that may come from i.e. coal gasification. However, I do not see the need to hide this high CO_2 selectivity just to further contribute to the hype of multifunctional systems for syngas to olefins. I do understand that previous works have (successfully) follow the same strategy to get published in high profile journals, but there has to be an end to it.

Apart from this point, there are other issues that the authors have to address:

The authors claim: "Given that the number of exposed 8-membered ring channels in AIPO-18 is higher than the SAPO-34 zeolite, a faster diffusion of reactants and products in AIPO-18 could be expected, which will likely affect the catalytic performance of bifunctional catalysts" This may be true, however, particles of both zeolites are very different in size, while primary AlPO-18 particles seem to be sub-nanometric, SAPO-34 crystals are all larger than 2-3 micron. Experiments with similar primary particles should be perform to further strengthen or withdraw this hypothesis

Effect of acidity on MTO like chemistry: the authors overlook a recent publication which I believe is relevant for the observed results: Nature Chem. 10 (2018) 804–812

In summary, this is an interesting paper that would be much more interesting if the authors focussed on highlighting challenges (CO_2 selectivity is a very important one) rather than in trying to sell their work as commercially viable when it probably is not yet there. In that line, performing experiments at different CO:H_2 ratios would be quite wise. It goes without saying that it is highly recommended to report not only CO conversion but also H_2 conversion.

Reviewer #2 (Remarks to the Author):

He and co-workers report on direct conversion of synthesis gas to lower olefins using a combination of ZnCr-oxide and low-Si AlPO-18 zeolite. By increasing reaction pressure they are able to achieve higher CO conversion levels than reported before. High selectivities to lower olefins and low selectivities to methane are obtained. This research can be considered as an important step to arrive at direct conversion of synthesis gas to lower olefins. Publication in NC is supported.

A number of comments are to be taken into account, however.

1. Under all circumstances high selectivities to CO_2 are apparent (Table S1) which limits the application of this process to CO-rich synthesis gas. If H_2-rich synthesis gas is used recycle of CO_2 will be necessary. These considerations should be mentioned in the main text.
2. General literature references 2-4 involve literature from the 1990-ies which is okay but should be

extended to more recent reviews and results, e.g., Angew. Chem. Int. Ed. 2016, 55, 2–4; ACS Catal. 2013, 3, 2130 –2149; Science 335, 835 (2012).
3. Mechanistic considerations (using DFT calculations) are restricted to hydride transfer. However, a lively debate is ongoing on the nature of the intermediate (methanol versus ketene) is ongoing in the literature. The authors should comment and contribute in this respect.
4. The stability reported in Figure 6 is promising. Next to conversion stability also catalyst coking should be considered. What are typical coke levels after catalysis?

Reviewer #3 (Remarks to the Author):

This manuscript reports a bifunctional catalyst for syngas to olefins and the catalyst consists of ZnCr binary oxide and low-Si AlPO-18 zeolite. Similar bifunctional catalysts have been reported in many previous papers including the previous work by the authors. The key claim of this work is the use of low Si AlPO-18 zeolite with AEI topology instead of SAPO-34 with CHA topology. The lower density and weaker strength of acid sites may be responsible for the high olefin selectivity of the current catalyst with low-Si AlPO-18 zeolites. The performance is quite good but not extraordinary. The high CO conversion of 40-50% can only be obtained at a very high syngas pressure (10 MPa). From the scientific sense, the insights offered by this work do not represent a significant advance in its field. Hence, this work lacks sufficient novelty/insights to meet the scientific level required by Nat. Commun. It is recommended that the manuscript is more appropriate for a specialized journal.

Reviewers' comments:

Reviewer #1 (Remarks to the Author):

This is an interesting manuscript that may have potential for Nature Communications but not in its current form.

First of all, the manuscript is misleading: all selectivities reported are in a CO2 free basis, considering that CO2 selectivities (only reported in the caption of figure 1) are in the order of a 50%, this means that selectivities to all the other products are actually half of those reported. When put in perspective, catalytic results are not far from those obtained when doing classical FTS chemistry on K promoted Fe catalysts (see for instance Nature Commun. 6 (2015) 6451). This is in principle fine, with CO:H2 ratios of 1, one would want to make CO2 to account for the H2 poor feed that may come from i.e. coal gasification. However, I do not see the need to hide this high CO2 selectivity just to further contribute to the hype of multifunctional systems for syngas to olefins. I do understand that previous works have (successfully) follow the same strategy to get published in high profile journals, but there has to be an end to it.

A: We agree with the reviewer's comments. The selectivity to CO_2, hydrocarbons and other products were recalculated, and updated in Figure 2 of the revised manuscript. Accordingly, the data in Figure 3 and Figure 6 were updated. In the revised manuscript, both the selectivity in the presence and absence of CO_2 were addressed in order to compare to the previous results.

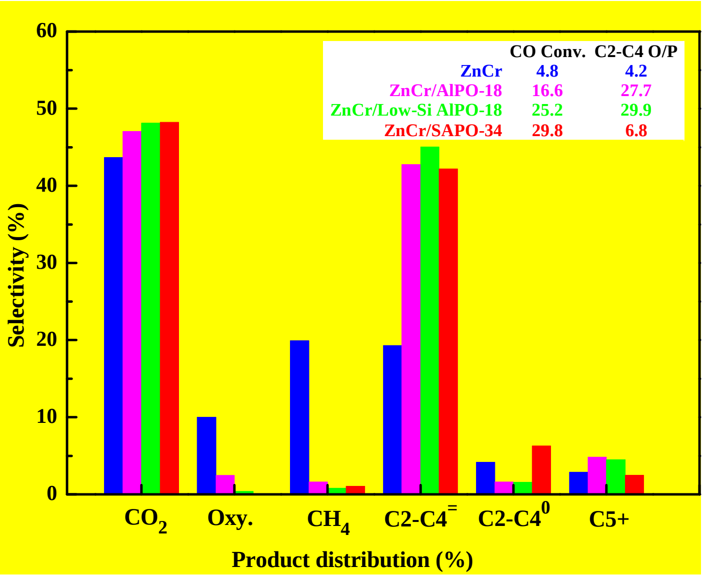

Figure 2. Catalytic performance of bifunctional catalyst containing ZnCr oxide and different zeolites. Reaction conditions: 390 °C, 4.0 MPa, 1200 h^{-1}, H_2/CO = 1, OX/ZEO = 1; Oxy.: Oxygenates including ethanol and methyl ether; C2-C4 O/P: C2-C4 olefin/paraffin ratio; C2-C4$^{=}$: C2-C4 olefins; C2-C4^{0}: C2-C4 paraffins; C5+: hydrocarbon products with more than 5 carbon.

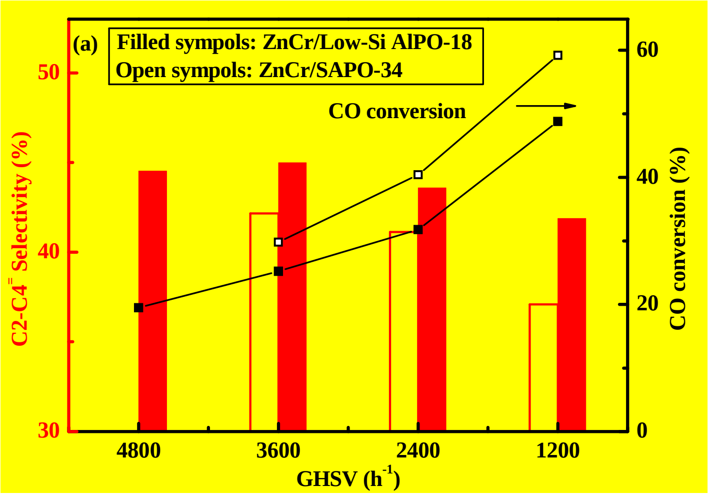

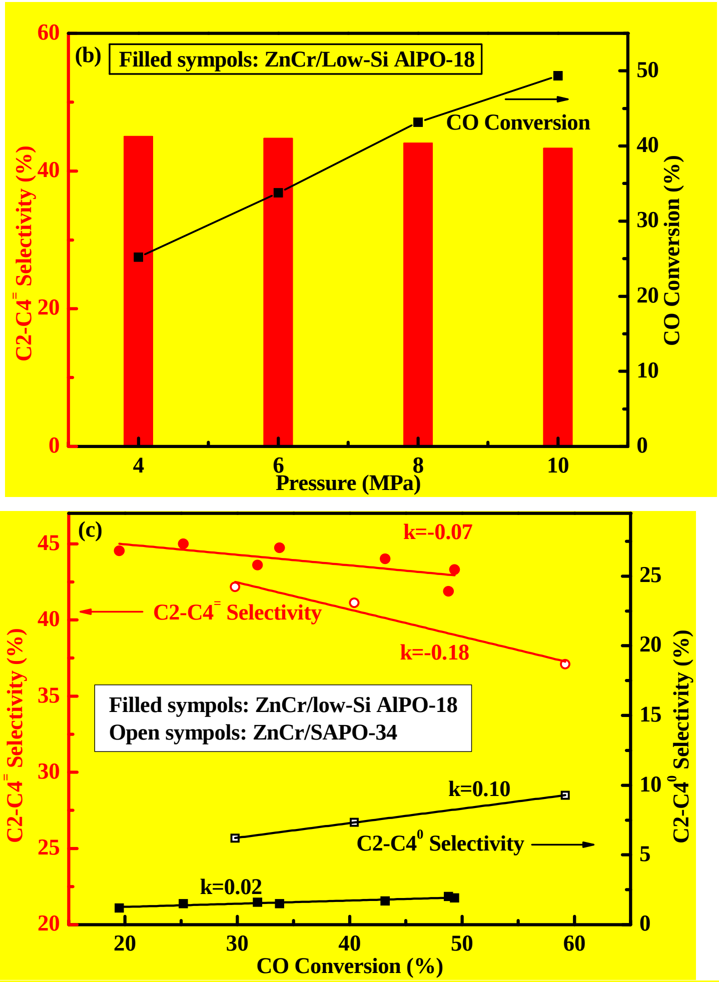

Figure 3. Effect of reaction conditions on catalytic performance. (a) Effect of GHSV on CO hydrogenation over ZnCr/ low-Si AlPO-18 and ZnCr/SAPO-34 bifunctional catalysts; Other conditions: 390 °C, 4.0 MPa, $H_2/CO = 1$; (b) Effect of pressure on CO hydrogenation over ZnCr/ low-Si AlPO-18 bifunctional catalysts; Other conditions: 390 °C, 3600 h^{-1}, $H_2/CO = 1$; (c) Relation between CO conversion and C2-C4 product selectivity. (ZnCr/low-Si AlPO-18: filled symbols, ZnCr/SAPO-34: open symbols).

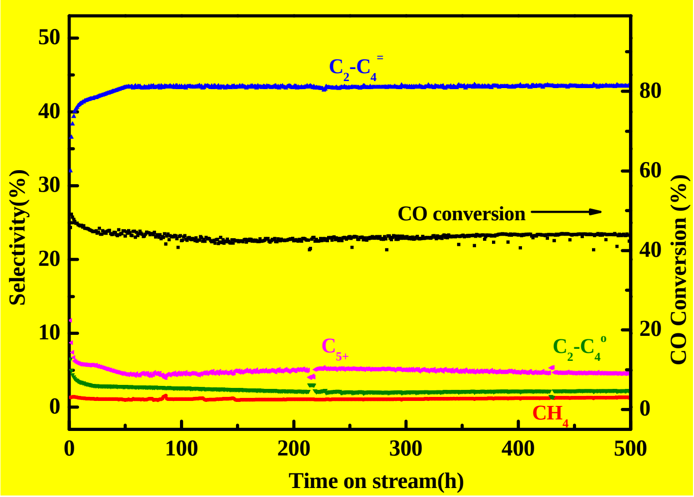

Figure 6. Stability test of ZnCr/ low-Si AlPO-18 bifunctional catalyst. Reaction conditions: 390 °C, 4.0 MPa, 1200 h^{-1}, H_2/CO = 1, and oxide/zeolite mass ratio = 1.

Apart from this point, there are other issues that the authors have to address:

The authors claim: "Given that the number of exposed 8-membered ring channels in AlPO-18 is higher than the SAPO-34 zeolite, a faster diffusion of reactants and products in AlPO-18 could be expected, which will likely affect the catalytic performance of bifunctional catalysts" This may be true, however, particles of both zeolites are very different in size, while primary AlPO-18 particles seem to be sub-nanometric, SAPO-34 crystals are all larger than 2-3 micron. Experiments with similar primary particles should be perform to further strengthen or withdraw this hypothesis

A: Yes, the particle size of zeolites is another important factor influencing the catalytic performance of bifunctional catalysts. Only using this kind of nanosheet low Si AlPO-18 zeolite can we obtain both high CO conversion and high selectivity to olefins. We synthesized a low Si AlPO-18 zeolite sample with lager particle size to investigate the size effect of zeolite on the reaction performance of the bifunctional catalysts, and the results were added in Figure S1 and Table S1 of the revised manuscript. We found that the zeolite particle size significantly affects CO conversion

and oxygenate selectivity, but slightly influences C2-C4 olefin/paraffin ratio. So in the revised manuscript, the following sentences *"a faster diffusion of reactants and products in AlPO-18 could be expected, which will likely affect the catalytic performance of bifunctional catalysts"* were deleted, and the following sentences on the particle size effect were added in page 6.

"The lower CO conversion and oxygenate selectivity imply that the driving force of AlPO-18 zeolite is insufficient for this reaction, possibly relevant with two aspects of the particle size and acidity. Regarding the former aspect, we further synthesized an AlPO-18 zeolite with 1-2 μm particle size (AlPO-18-L, Figure S1) to investigate the size effect of zeolite on the catalytic performance (Table S1). The ZnCr/AlPO-18-L shows even lower CO conversion (8.8%) and a higher selectivity to oxygenates (DME and MeOH, 30.8%). However, it is noteworthy that although the total selectivity of hydrocarbons decreases, the C2-C4 olefin/paraffin ratio remains at a level of 27.5, which means that the particle sizes of zeolite does not show significant effect on hydrocarbon distribution."

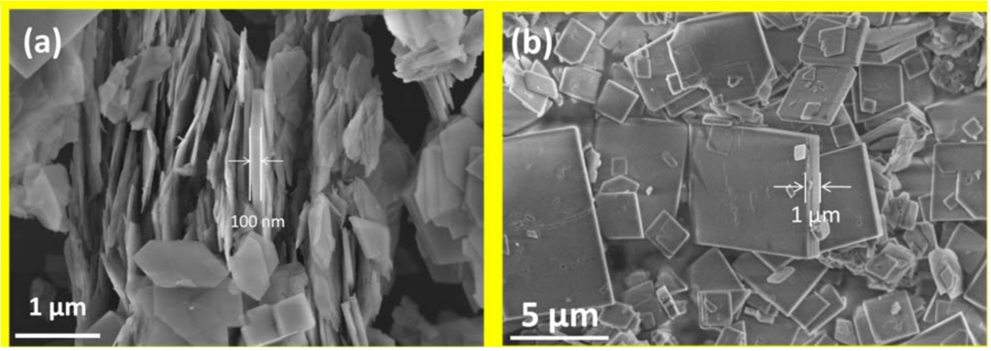

Figure S1. SEM images of the calcined AlPO-18(a) and AlPO-18-L(b).

Table S1. Catalytic performance of bifunctional catalyst containing ZnCr oxide and different zeolites.

Catalyst	*CO conv. (%)*	*H_2 conv. (%)*	*Selectivity (%)*			*Hydrocarbon distribution[c] (%)*				*C_{2-4} O/P ratio*
			CO_2	*HC.*	*Oxy.[d]*	*CH_4*	*$C_{2-4}^{=}$*	*C_{2-4}^{0}*	*C_{5+}*	
ZnCr[a]	*4.8*	*4.4*	*43.7*	*46.3*	*10.0*	*43.0*	*41.0*	*9.7*	*6.3*	*4.2*
ZnCr/AlPO-18[b]	*16.6*	*10.3*	*47.0*	*50.6*	*2.4*	*3.0*	*84.4*	*3.0*	*9.6*	*27.7*
ZnCr/low-Si AlPO-18[b]	*25.2*	*14.6*	*48.1*	*51.6*	*0.3*	*1.4*	*86.7*	*2.9*	*8.9*	*29.9*
ZnCr/low-Si AlPO-34[b]	*24.9*	*14.5*	*48.2*	*51.8*	*0.0*	*1.9*	*84.4*	*7.9*	*5.8*	*10.5*
ZnCr/SAPO-34[b]	*29.8*	*17.6*	*48.2*	*51.8*	*0.0*	*1.9*	*81.5*	*12.0*	*4.6*	*6.8*
ZnCr/AlPO-18-L[b]	*8.8*	*7.2*	*39.9*	*29.3*	*30.8*	*3.4*	*86.0*	*3.1*	*7.5*	*27.5*

[a] reaction conditions: 390 °C, 4.0 MPa, 1200 h^{-1}, H_2/CO = 1, OX/ZEO = 1; [b] reaction conditions: 390 °C, 4.0 MPa, 3600 h^{-1}, H_2/CO ratio = 1, OX/ZEO = 1; [c] Hydrocarbon distribution was calculated based on carbon molar amount without CO_2 and Oxygenates; [d] include methanol and methyl ether.

Effect of acidity on MTO like chemistry: the authors overlook a recent publication which I believe is relevant for the observed results: Nature Chem. 10 (2018) 804–812

A: Thanks for this suggestion. We read carefully this recommended paper and found that the findings in this paper highly support the issue of the effect of acidity of our manuscript. We added a discussion in page 10 of the revised manuscript. *"Interestingly, previous work[31] have shown that the isolation of Brønsted acid sites is beneficial to maximize olefin selectivity by preventing secondary reactions in MTO*

process. In this aspect, the decrease of acid density also helps to promote the acid site isolation. Considering the catalytic difference in SAPO-34 and low-Si AlPO-34 zeolites, we demonstrated that reducing the acid density of zeolites and/or promoting the isolation of acid sites is beneficial to slightly increase the olefin/paraffin ratio, consistent with previous work[19,20,31]*.*"

In summary, this is an interesting paper that would be much more interesting if the authors focussed on highlighting challenges (CO2 selectivity is a very important one) rather than in trying to sell their work as commercially viable when it probably is not yet there. In that line, performing experiments at different CO:H2 ratios would be quite wise. It goes without saying that it is highly recommended to report not only CO conversion but also H2 conversion.

A: We highly appreciate the reviewer's comments considering this is an interesting paper. We deleted related sentences on the commercially viable and then highlighted the scientific challenges in syngas to olefins conversion including CO_2 selectivity. The detail as follows:

To delete: *"The coupling of several cascade reactions into a direct process using one reactor could break the inherent thermodynamic equilibrium limit of each individual reaction and improve the overall conversion efficiency. From the perspective of industrial applications, this could simplify the reaction-separation process and reduce the investment cost."* in page 2.

To delete: *"It is generally accepted that the reaction pathway mainly consists of two steps: the initial hydrogenation of CO over oxide component and the following C-C coupling over zeolite component. It was previously suggested that ketene or methanol may be the key intermediate produced from oxide and then transformed into light olefins in zeolites.*[19,20,23] *Interestingly, as the selectivity of light olefins is very similar to that of the MTO conversion, this direct process could be considered as a promising alternative with high potential industrial application to produce light olefins from coal or natural gas to circumvent the drawback of the MTO conversion."* in page 3.

To add: "*Despite the great progress made previously, the industrialization of the direct conversion is still impeded by several major challenges. First, the CO conversion should be significantly improved to decrease the energy consumption for the separation of unconverted syngas from light olefins products. Second, the effective utilization of carbon resources is low as the result of undesired* CO_2 *production; currently, either modified FT catalysts*[24,25]*, or bifunctional catalysts*[19-23]*, both show a* CO_2 *selectivity of higher than 45%. Indeed, it is one of the major challenges especially for the conversion of hydrogen rich syngas derived from nature gas. However, for the conversion of hydrogen lean syngas derived from coal or biomass, it's necessary to modulate* H_2/CO *ratio via water-gas shift reaction. Consequently, high* CO_2 *selectivity can enable the in-situ re-adjustment of the* H_2/CO *molar ratio, which is benefit for coal- or biomass-based syngas conversion. Third, among the* CO_2 *free products, it is very necessary to enhance the selectivity to the desired products of light olefins while reduce the selectivity to paraffins and maintain high CO conversion. The maximal CO conversion of the direct STO process using bifunctional catalysts is reported to be only ~17%*[19]*. As olefins may be further hydrogenated into paraffins in the presence of hydrogen*[23]*, the selectivity of C2-C4 paraffins would be increased when enhancing the CO conversion. So it is very challenging to simultaneously achieve high CO conversion and selectivity to light olefins in the direct STO conversion.*" in page 3.

Besides, the catalytic activity over ZnCr/low-Si AlPO-18 with different H_2/CO mole ratio was tested and listed in Table S3 of the revised SI. The H_2 conversion was included and the corresponding discussion was added as can be seen in page 7.

"In addition, H_2/CO *mole ratio is another effect on this conversion. As shown in Table S3, with an increase in* H_2/CO *mole ratio, the CO conversion rises while the* H_2 *conversion drops obviously. As high* H_2 *concentration may facilitate the olefins hydrogenation and reverse water-gas shift reaction, our results show gradual decrease in the selectivity to C2-C4 olefins and* CO_2 *as* H_2/CO *increases despite of the increase of carbon utilization (selectivity to hydrocarbons)."*

Table S3. Effect of H_2/CO on CO hydrogenation over ZnCr/ low-Si AlPO-18 bifunctional catalyst[a]

H_2/CO	CO conv. (%)	H_2 conv. (%)	Selectivity (%)			Hydrocarbon distribution[b] (%)				C_{2-4} O/P ratio
			CO_2	HC.	Oxy. [c]	CH_4	$C_{2-4}^{=}$	C_{2-4}^{0}	C_{5+}	
0.5	29.5	39.9	45.0	54.7	0	1.5	82.5	2.2	13.8	39.3
1.0	48.8	29.9	47.2	52.6	0.1	1.3	80.7	4.0	14.0	20.7
2.0	68.7	26.7	42.0	58	0	1.4	77.2	7.8	13.4	10.0
4.0	70.7	16.3	38.2	61.8	0	2.4	67.5	16.5	13.6	4.1

[a] reaction conditions: 390 °C, 4.0 MPa, 1200 h^{-1}, OX/ZEO = 1; [b] Hydrocarbon distribution was calculated based on carbon molar amount without CO_2 and Oxygenates; [c] include methanol and methyl ether.

Reviewer #2 (Remarks to the Author):

He and co-workers report on direct conversion of synthesis gas to lower olefins using a combination of ZnCr-oxide and low-Si AlPO-18 zeolite. By increasing reaction pressure they are able to achieve higher CO conversion levels than reported before. High selectivities to lower olefins and low selectivities to methane are obtained. This research can be considered as an important step to arrive at direct conversion of synthesis gas to lower olefins. Publication in NC is supported.

A number of comments are to be taken into account, however.

1. Under all circumstances high selectivities to CO2 are apparent (Table S1) which limits the application of this process to CO-rich synthesis gas. If H2-rich synthesis gas is used recycle of CO2 will be necessary. These considerations should be mentioned in the main text.

A: Thanks for the suggestion. The following sentences were added in the revised manuscript to discuss the effect of CO_2 selectivity on application of this process to H_2-rich syngas and CO-rich (H_2 lean) syngas in page 3.

"*Second, the effective utilization of carbon resources is low as the result of undesired CO_2 production; currently, either modified FT catalysts*[24,25]*, or bifunctional catalysts*[19-23]*, both show a CO_2 selectivity of higher than 45%. However, for the conversion of hydrogen lean syngas derived from coal or biomass, it's necessary to modulate H_2/CO ratio via water-gas shift reaction. Consequently, high CO_2 selectivity can enable the in-situ re-adjustment of the H_2/CO molar ratio, which is benefit for coal- or biomass-based syngas conversion.*"

2. General literature references 2-4 involve literature from the 1990-ies which is okay but should be extended to more recent reviews and results, e.g., Angew. Chem. Int. Ed. 2016, 55, 2–4; ACS Catal. 2013, 3, 2130 –2149; Science 335, 835 (2012).

A: These recent reviews and articles were cited in the revised manuscript.

3. Mechanistic considerations (using DFT calculations) are restricted to hydride transfer. However, a lively debate is ongoing on the nature of the intermediate (methanol versus ketene) is ongoing in the literature. The authors should comment and contribute in this respect.

A: Thanks very much for pointing this very interesting topic. Both methanol and ketene were once suggested as the key intermediate produced on oxides and transformed into light olefins in zeolites. We previously proposed that the ketene conversion to light olefins (KTO) proceeds via the hydrocarbon pool mechanism as in the MTO conversion based on theoretical calculations. Then H_2O or CO were respectively left as by-product in the KTO or MTO processes. By comparing the kinetics of the chain propagation using olefin methylation as example, we found that

KTO is less active than MTO. More importantly, relatively large amount of oxygenates (MeOH and DME) were observed in pure oxide component despite of low CO conversion of this work. Based on these findings, we speculated that it is methanol other than ketene acts as the key intermediate linking oxide and zeolite components. The mechanistic section was revised accordingly to include the above mentioned consideration. It should be mentioned that the ketene chemistry in zeolites should be studied systematically and separately as recently suggested by Gason et al. (Angew. Chem. Int. Ed. 2018, 57, 14982 – 14985).

To add: *"In the bifunctional catalysts for the direct conversion of syngas to light olefins, methanol and/or ketene were previously suggested as the key intermediate produced on oxides and transformed in zeolites to olefins[19,20]. We have theoretically proved that the ketene to olefins conversion proceeds via the hydrocarbon pool mechanism, similar to the MTO conversion in zeolites where CO or H_2O were respectively formed in both cycles[32]. Therefore, the selectivity of olefins mainly correlates with the distribution of cracking precursors, highly affected by the structure of zeolites (e.g. acidity, topology) and reaction conditions[2,33]."* and "Regarding the intermediate species, we speculated that methanol is likely to play a greater role than ketene, since relatively larger amounts of oxygenates (methanol or DME) are produced on pure ZnCr oxides and ketene is less reactive than methanol for the chain propagation in the hydrocarbon pool mechanism[32,34]." in page 13.

4. The stability reported in Figure 6 is promising. Next to conversion stability also catalyst coking should be considered. What are typical coke levels after catalysis?

A: We agree with reviewer's suggestion. The TGA curves of used ZnCrOx oxide and low-silica AlPO-18 zeolite were included in Figure S3. We also added the discussion part in page14.

"The TGA curves (Figure S2) of used catalysts shows that there are 1.5% and 15% weight loss in ZnCr oxide and low-Si AlPO-18 zeolite respectively, which contain volatile species and occluded organic deposits. After removal of volatile components

in N_2 flow, 3.5% of carbon deposit amount in zeolite is obtained by measuring the amount of CO_2 formed from the combustion of the used bifunctional catalyst*.”*

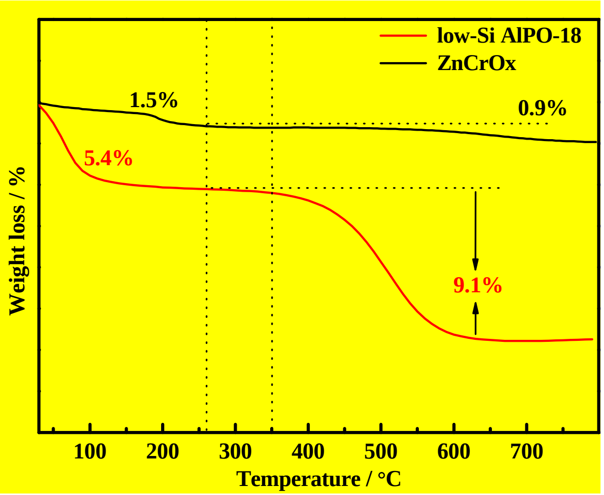

Figure S2. TGA curves of used ZnCrOx oxide and low-silica AlPO-18 zeolte obtained after syngas conversion at 673 K.

Reviewer #3 (Remarks to the Author):

This manuscript reports a bifunctional catalyst for syngas to olefins and the catalyst consists of ZnCr binary oxide and low-Si AlPO-18 zeolite. Similar bifunctional catalysts have been reported in many previous papers including the previous work by the authors. The key claim of this work is the use of low Si AlPO-18 zeolite with AEI topology instead of SAPO-34 with CHA topology. The lower density and weaker strength of acid sites may be responsible for the high olefin selectivity of the current catalyst with low-Si AlPO-18 zeolites. The performance is quite good but not extraordinary. The high CO conversion of 40-50% can only be obtained at a very high syngas pressure (10 MPa). From the scientific sense, the insights offered by this work do not represent a significant advance in its field. Hence, this work lacks

sufficient novelty/insights to meet the scientific level required by Nat. Commun. It is recommended that the manuscript is more appropriate for a specialized journal.

A: Thanks for the reviewer's comments. It is true that the concept of bifunctional catalyst firstly proposed by Bao and coworkers has attracted much considerable interests, and many bifunctional catalysts were then successfully prepared and tested for the CO or CO_2 conversion into hydrocarbons with high selectivity (such as light olefins, paraffins, aromatics) (Angew. Chem. Int. Ed., 2016, 55, 4725-4728. Angew. Chem. Int. Ed., 2018, 57 4692-4696. Chem. Sci., 2018, 9, 4708-4718. Chem, 3 (2017) 334-347. Nat Chem, 9 (2017) 1019-1024…..). Very recently, we conducted similar research work integrating oxide and SAPO-34 for the STO conversion (ChemCatChem, 2018, 10, 1536-1541). Similar to those reported data in references, we found that it is extremely difficult to simultaneously improve CO conversion and maintain high selectivity to C2-C4 olefin (seesaw-type effect), which is particularly important in industrial catalysis. Aiming to a special type of products, like paraffins, aromatics, the zeolite components were selected using ZSM-5 zeolites. But in the case of the CO conversion into light olefins (C2-C4 olefins), we found that the attention were mainly focused on tailoring or optimizing the oxide components. It should be mentioned that SAPO-34 zeolite is the best choice for the MTO conversion, but it may not be the best choice in the bifunctional catalysts for the STO conversion to light olefins. According to the understanding that oxide component mainly contributes to the conversion of CO while zeolite component mainly dedicates to the product distribution, we believe that the above-mentioned seesaw-type effect between CO conversion and selectivity to light olefins could be alleviated by modulating zeolite component, which is the key content of this manuscript for developing novel bifunctional catalytic systems to light olefins.

In this work, our research direction is quite different from previous studies. We designed a novel bifunctional catalyst containing zeolite component with AEI structure, and simultaneously achieved high CO conversion (~50%) and C2-C4 olefin selectivity (> 80%) with an unprecedented high olefin ratio (> 20), which makes the bifunctional catalyst more competitive in industrialization. By in situ characterizations

of acidity and DFT calculations, we systematically studied the correlation between the formation of paraffin by-products and the properties (acidity and cage structure) of small porous zeolite in the direct synthesis of olefins from syngas. Based on these results, we proposed that hydrogen transfer in zeolites is one of the important ways dedicating to the paraffins formation and the zeolite with weak performance (less acid sites, weak acidity strength) would be much better than those with strong performance. We believe that this scientific insight (less or weak is better) would offer anther different idea for the development of novel bifunctional catalysts. The novelty and importance of this manuscript, we believe, are tenable.

REVIEWERS' COMMENTS:

Reviewer #1 (Remarks to the Author):

The authors have addressed the most important criticism raised during the first round of review. I believe that the work is now much stronger and information given is much clearer. I support publication in its current form.

Reviewer #2 (Remarks to the Author):

The authors have significantly improved the manuscript by responding and acting upont the reviewers'comments. I support publication in NatComm.
